# Supplementary material for: Appraisal of published guidelines in European countries addressing the clinical care of childhood sexual abuse: protocol for a systematic review
Source: BMJ Open. 2023 Apr 17;13(4):e064008. doi: 10.1136/bmjopen-2022-064008 (PMC10111900; doi:10.1136/bmjopen-2022-064008)
Supplement: Supplementary data [file bmjopen-2022-064008supp001.pdf]

Appendix to revised Manuscript ID bmjopen-2022-064008.R1

Updated search strategy_CSA_Guidelines_SR_MEDLINE format_14_04_2022_Final

1. Child abuse.mp. or Child Abuse/
2. Abused child.mp.
3. Child sexual abuse.mp. or Child Abuse, Sexual/
4. Child sexual exploitation.mp.
5. (sexual violence or sexual assault or rape or sex trafficking or sexual coercion).mp.
6. or/1-5
7. Diagnosis/ or Diagnosis, Differential/ or diagnosis.mp.
8. diagnostic workup.mp.
9. investigation.mp.
10. (management or management tool or medical management or assessment).mp.
11. (critical pathway or clinical pathway or care path or critical path or toolkit).mp.
12. or/7-11
13. (teen or teens or teenage*).mp.
14. (adolesc* or preadolesc* or pre-adolesc* or juvenil*).mp.
15. (youth or youths or youngster*).mp.
16. ((young adj (person* or persons or people)) or early adult*).mp.
17. (student or students or schoolchild*).mp.
18. exp infant/
19. exp Child/
20. Young adult/
21. adolescent/
22. (boy* or girl* or child or children or infant or infants or kid or kids).mp.
23. (pediatri* or paediatri*).mp
24. or/13-23
25. Guideline Adherence/ or guidelines.mp. or Guideline/ or Practice Guideline/
26. clinical guideline.mp.
27. recommendation.mp.
28. recommendation*.mp.
29. guideline*.mp.
30. clinical gudeline*.mp.
31. or/25-30
32. 6 and 12 and 24 and 31
33. limit 32 to yr="2012-2022"

CSA_SR_Search strategy for other databases_free keyword format

(child abuse or child sexual abuse or child sexual exploitation or sexual violence or sexual assault or rape or sex trafficking or sexual coercion)

AND (Diagnosis or diagnostic work up or investigation or management or assessment or clinical pathway or care path or critical pathway)

AND (Infant or child or young adult or adolescent or teenager or boy or girl) AND (Clinical guideline or guideline or practice guideline or recommendation)
