# Supplementary material for: Appraisal of published guidelines in European countries addressing the clinical care of childhood sexual abuse: protocol for a systematic review
Source: BMJ Open. 2023 Apr 17;13(4):e064008. doi: 10.1136/bmjopen-2022-064008 (PMC10111900; doi:10.1136/bmjopen-2022-064008)
Supplement: Author's manuscript [file bmjopen-2022-064008.draft_revisions.pdf]

BMJ Open

BMJ Open is committed to open peer review. As part of this commitment we make the peer review history of every article we publish publicly available.

When an article is published we post the peer reviewers' comments and the authors' responses online. We also post the versions of the paper that were used during peer review. These are the versions that the peer review comments apply to.

The versions of the paper that follow are the versions that were submitted during the peer review process. They are not the versions of record or the final published versions. They should not be cited or distributed as the published version of this manuscript.

BMJ Open is an open access journal and the full, final, typeset and author-corrected version of record of the manuscript is available on our site with no access controls, subscription charges or pay-per-view fees (http://bmjopen.bmj.com).

If you have any questions on BMJ Open's open peer review process please email info.bmjopen@bmj.com

BMJ Open

Appraisal of Published Guidelines in European Countries Addressing the Clinical Care of Childhood Sexual Abuse: Protocol for a Systematic Review.

Journal:	*BMJ Open*
Manuscript ID	bmjopen-2022-064008
Article Type:	Protocol
Date Submitted by the Author:	21-Apr-2022

Complete List of Authors:	Otterman, Gabriel; Linköping University, Department of Biomedical and Clinical Sciences<br>Nurmatov, Ulugbek; Division of Population Medicine, School of Medicine, Cardiff University<br>Naughton, Aideen; Cardiff University,<br>Kemp, Alison; Department of Child Health, Division of Population Medicine<br>Korhonen, Laura; Linköping University, Biomedical and Clinical Sciences<br>Karst, Wouter; Nederlands Forensisch Instituut, Forensic Medicine<br>Jud, Andreas; University Hospital Ulm<br>Vollmer Sandholm, Mary Jo; Oslo University Hospital<br>Mora-Theuer, Eva; Medical University of Vienna<br>Moultrie, Sarah; Children's Hospital Colorado<br>Akhlaq, Ather; Institute of Business Management ,<br>Chalumeau, Martin; Necker-Enfants Malades Hospitals, Department of General Pediatrics and Pediatric Infectious Diseases<br>Greenbaum, Jordan; International Centre for Missing & Exploited Children

Keywords:	Child protection < PAEDIATRICS, Protocols & guidelines < HEALTH SERVICES ADMINISTRATION & MANAGEMENT, Quality in health care < HEALTH SERVICES ADMINISTRATION & MANAGEMENT, ACCIDENT & EMERGENCY MEDICINE

SCHOLARONE™
Manuscripts

Title: Appraisal of Published Guidelines in European Countries Addressing the Clinical Care of Childhood Sexual Abuse: Protocol for a Systematic Review.

Word count: 1,645

Section 1: Administrative Information:

Registration: In accordance with PRISMA-P guidelines, our systematic review protocol was registered with the International Prospective Register of Systematic Reviews (PROSPERO)[1] on registration number: (CRD42022320747).

Authors:

Otterman GJ[1], Nurmatov UB[2], Naughton A[2], Kemp A[2], Korhonen L[1], Karst WA[3], Jud A[4], Vollmer Sandholm MJ[5], Mora Theuer EA[6], Moultrie S[7], Akhlaq A[8], Chalumeau M[9], Greenbaum J[10] and the COST Action 19106 Research Team.

Affiliations:
[1]Linköping University, Linköping, Sweden
[2]Cardiff University, Wales, United Kingdom
[3]Netherlands Forensic Institute, The Hague, Netherlands
[4]Ulm University Hospital, Ulm, Germany
[5]Oslo University Hospital, Oslo, Norway
[6]Medical University of Vienna, Vienna, Austria
[7]Children's Hospital Colorado (formerly), Aurora, United States of America
[8]Institute of Business Management, Karachi, Pakistan
[9] Necker-Enfants Malades Hospital, Université de Paris, Paris, France
[10]International Centre for Missing and Exploited Children, Alexandria, United States of America

Corresponding author:

Name of the corresponding author:	Gabriel J. Otterman
Affiliation:	Barnafrid National Centre on Violence Against Children<br>Department of Biomedical and Clinical Sciences<br>Linköping University<br>SE-58183 Linköping<br>Sweden
Email:	gabriel.otterman@liu.se

For peer review only

ABSTRACT:

Introduction: Child sexual abuse (CSA) is a global public health problem with potentially severe health and mental health consequences. Healthcare professionals (HCPs) should be familiar with risk factors and potential indicators of CSA, and able to provide appropriate medical management. The World Health Organization (WHO) issued guidelines for the clinical care of children with CSA for the international community, based on rigorous review of the evidence base. The current systematic review identifies existing CSA guidelines issued by government agencies and academic societies in the European Region and assesses their quality and clarity to illuminate strengths and identify opportunities for improvement.

For peer review only - http://bmjopen.bmj.com/site/about/guidelines.xhtml

Methods and analysis: This 10-database systematic review is guided by the Centre for Reviews and Dissemination and The Preferred Reporting Items for Systematic reviews and Meta-Analyses (PRISMA) statement. Guidance for HCPs regarding CSA, written by a national governmental agency or academic society of health professionals within 34 COST Action 19106 Network Countries (CANC) and published in the peer-reviewed or grey literature between Jan. 1, 2012-Jan 1, 2022, are eligible for inclusion. Two independent researchers will search the international literature, screen, review and extract data. Included guidelines will be assessed for completeness and clarity, compared to the WHO 2017/2019 guidelines on CSA, and evaluated for consistency between the CANC guidelines. The Appraisal of Guidelines for Research and Evaluation (AGREE II) tool and the Grading of Recommendations Assessment, Development, and Evaluation (GRADE) working group methodology will be used to evaluate CANC guidelines. Descriptive statistics will summarise content similarities and differences between the WHO guidelines and national guidelines; data will be summarised using counts, frequencies, proportions and percent agreement between country-specific guidelines and the WHO 2017 guidelines.

Ethics and dissemination: There are no individuals or protected health information involved and no safety issues identified. Results will be published in a peer-reviewed medical journal.

Registration details: PROSPERO #: CRD42022320747.

Strengths and Limitations:

Strengths:

- This review will assess the quality and clarity of CSA guidelines issued by government agencies and academic societies in the European Region to inform future efforts to improve and strengthen the knowledge of HCPs and their response to CSA. The reference standards for comparison are two international, evidence-based guidelines published by the World Health Organization.[2,3]
- This systematic review will for the first time evaluate the quality and consistency of CSA clinical guidelines across European countries.
- An international team of subject-matter experts in the field of child abuse and neglect in collaboration with experts in systematic review methodology will conduct this study using a robust and transparent approach.

Limitations:

- The different methods used in developing the guidelines and formulating recommendations across a diversity of practice settings may underpin the variability of both scope and quality of guidelines.
- Recognising that in some European countries, healthcare is organised at the state, provincial or municipal level, our search strategy that is limited in scope to a national level may miss guidelines that are used in practice.

INTRODUCTION

Rationale:

Childhood sexual abuse (CSA) encompasses a spectrum of inappropriate and (potentially) criminal sexual activities involving children and youth under 18 years of age.[4] Rates of CSA are generally high across the globe, with prevalence estimates ranging from 3.7% to 40% in European countries.[5] Adverse physical

For peer review only - http://bmjopen.bmj.com/site/about/guidelines.xhtml

health, mental health and societal consequences of CSA include infection (e.g., HIV, syphilis and other sexually transmitted infections [STIs]); anogenital injury; unwanted pregnancy; substance abuse disorders; post-traumatic stress disorder (PTSD); major depression, often associated with self-harm and suicidal ideation; anxiety disorders and behavioural challenges, including antisocial behavior.[6,7] As a result of these health conditions, many children and youth who experience one or more forms of CSA seek care from health professionals. They may present for acute care after a specific event or may seek care for other reasons which may or may not be related to their victimisation. In the latter case, patients may not disclose their status and the healthcare professional (HCP) must be alert to potential risk factors and indicators of CSA.[8] In addition, the clinician must be knowledgeable about the appropriate set of responses in the care and follow-up of CSA and skilled at providing care using a trauma-informed, patient-centred approach.[9] Training of HCPs on CSA is often lacking in Europe and elsewhere.[10-13] Similarly, medical and nursing students are not routinely taught about trauma-informed, patient-centred care, which involves recognising the potential impact of prior traumatising events on a patient, caregiver and others, and responding with empathy and support. With a trauma-informed approach the HCP makes every effort to minimize patient and caregiver distress during the visit, to actively involve these individuals in discussions and decisions, to prioritise their needs and facilitate short and long-term healing. [14]

To assist HCPs in recognising and appropriately responding to CSA, healthcare organisations need specific protocols and guidelines providing detailed information that aims to optimise care and maximise efficiency. Based upon rigorous review of the global scientific literature on childhood sexual violence,[15] the World Health Organization (WHO) published evidence-based, comprehensive clinical guidelines in 2017 and 2019.[2,3] Countries may rely on these documents to guide their HCPs or base their national guidelines on WHO content. National and regional medical academic societies also publish guidelines.[16-20] However, the nature of CSA guidelines varies in content and detail. HCPs in many countries do not have access to guidelines addressing CSA, due to lack of resources at the local, national or geographic regional level and lack of awareness of the WHO guidelines. This may lead to differences across countries and regions in access to evidence-based medical care for vulnerable children and youth.

It is important to identify existing CSA guidelines issued by government agencies and academic societies in the European Region and assess their quality and clarity in order to illuminate strengths and identify opportunities for improvement. This will help drive a targeted approach to enhancing the healthcare response to CSA and facilitate comprehensive protocol development across the European Region. We posit that disparities in healthcare and health-related outcomes for survivors will decrease as quality of care is increased across the European countries.

Objectives:

The aim of this systematic review is to critically appraise existing clinical guidelines addressing the clinical care of CSA published in the COST Action 19106 Network Countries (CANC).[21] The review will address the following questions:

1. In comparison to the World Health Organization's 2017 and 2019 clinical guidelines on the health sector response to child sexual abuse, what are the completeness, clarity and consistency of healthcare guidelines published by countries within the European Region addressing CSA?
2. How do the included CANC countries guidelines compare with one another in content consistency?

For peer review only - http://bmjopen.bmj.com/site/about/guidelines.xhtml

METHODS

This systematic review will be performed according to guidance from the Preferred Reporting Items for Systematic reviews and Meta-Analyses (PRISMA) statement.[22]

Eligibility criteria: Guidelines will be eligible for inclusion in the systematic review if they meet all the following criteria:

- Include specific guidance for healthcare professionals to recognise and appropriately respond to children (<18 years) who have experienced CSA
- Are published in peer-reviewed journals or in the grey literature (including government or health professional society websites)
- Represent the product of a national governmental agency or an academic society of health professionals located within the CANC
- Are published between Jan 1, 2012, through Jan 1, 2022 (reports published prior to this time may be outdated in certain topics such as diagnosis and treatment of infection)
- No language restrictions

Information sources

Literature search: The systematic review will entail a search of CINAHL, EMBASE, Google Scholar, Guidelines International Network, Medline, PsycINFO, SUMSearch, TRIP, Web of Science, and WHO Global Health Library, using the Google search engine. References of the guidelines included in the review will be searched for additional guidelines. Search of the grey literature will include the Health Management Information Consortium (HMIC), the National Institute for Health and Care Excellence (NICE, UK), as well as the websites of government health agencies and academic societies within the European Region. In addition, members of the Multi-sectoral Responses to Child Abuse and Neglect in Europe (Euro-CAN; COST Action 19106, a network of experts in child maltreatment and relevant stakeholders representing 34 countries) will be contacted and asked to identify additional relevant guidelines. Paediatricians, forensic physicians, gynaecologists and other physicians in the CANC engaged in the care of vulnerable children will be included in the search using a snowball strategy.

Search strategy: Search terms will be developed under the expert guidance of one of the research team leads (UN) using medical subject headings (MeSH) and text words related to forms of childhood sexual abuse, and healthcare professional guidance. A draft of a Medline search strategy is included in Appendix 1. After finalisation of the Medline search strategy criteria, the latter will be adapted for the remaining electronic databases. The time period and language restrictions for guideline inclusion are described above.

Patient and public involvement statement: Patients and the public were not involved in this systematic review of published national guidelines.

Study records

Data management: Retrieved studies from all international electronic databases will be imported into EndNote software and de-duplicated, before being screened and identified as eligible guidelines. Completed screening records, and full texts of included studies will be stored as a Master Database in EndNote software for evaluation.

Selection process: Two authors will search databases and screen titles and abstracts independently for potentially eligible studies. Disagreement between researchers will be resolved by consensus or

For peer review only - http://bmjopen.bmj.com/site/about/guidelines.xhtml

arbitration involving a third author where necessary. Full texts of studies will be retrieved for selected guidelines, and two authors will evaluate whether these meet inclusion/exclusion criteria. Disagreement will be resolved by referral to a third author if necessary. Reasons for the excluded papers will also be provided. The list of final selected studies will be shared with experts (max. 3) for any missing or on-going/unpublished studies.

Data collection process

Two independent reviewers will extract data from included guidelines and enter it into a customised data extraction sheet. The two sheets will be compared for consistency and discrepancies resolved as above. The resultant data will be entered into the master database (Microsoft Excel). Non-English guidelines will be translated using readily available tools such as Google Translate and by consulting individuals who are bilingual, including in that particular language.

Data items: The customised data extraction sheet will include the following information: country, year of dissemination, guideline development process with the report of the group membership involved, search method, and specific content and characteristics of guidelines as they relate to the good practice statements and recommendations from the "Responding to children and adolescents who have been sexually abused: WHO 2017 clinical guidelines"[2] and the sections on identification and prevention of recurrence of CSA included in the "WHO Guidelines for the Health Sector Response to Child Maltreatment: Technical Report, 2019."[3] Examples of content and characteristics to be assessed and extracted include components of trauma-informed care, and of HIV post-exposure prophylaxis.

Outcomes and prioritisation:
Primary outcome(s):
Alignment with WHO guidelines.[2,3] Completeness, clarity and consistency of national guidelines compared to reference standard.
Secondary outcomes:
Consistency between the included clinical guidelines of the CANC

Risk of bias in individual studies: Eligible guidelines will be critically appraised by two or more independent reviewers working in duplicate. The quality, methodological rigour and transparency of included clinical guidelines will be assessed using Appraisal of Guidelines for Research & Evaluation AGREE II tool.[23,24]

Data synthesis: Descriptive statistics will be employed to characterise content similarities and differences between the reference standard "Responding to children and adolescents who have been sexually abused: WHO 2017 clinical guidelines" and included national guidelines for the diagnosis of child sexual abuse issued in European countries. Key findings from each guideline will be summarised and presented in descriptive summary tables. Data will be reported using counts, frequencies and proportions (e.g., the proportion of guidelines that address a trauma-informed approach). Members of the research team will extract key quality indicators from the WHO 2017 guideline. Each country-specific guideline will be examined for the presence of the indicators. The percent agreement on the rating of country-specific guidelines will be calculated and presented. We will summarise the findings by providing a systematic narrative synthesis with the information presented in text and tables.

Meta-biases: No assessment for meta-bias is planned for this protocol review.

For peer review only - http://bmjopen.bmj.com/site/about/guidelines.xhtml

Confidence in cumulative estimate: The Grading of Recommendations Assessment, Development, and Evaluation (GRADE) working group methodology will be used to assess the clarity and strength of underlying evidence inside guideline statements. Additionally, the scope description and grade or rating of the guideline recommendations will be assessed.[25]

Ethics and dissemination: The systematic review does not involve individuals or protected health information and there are no safety issues identified. Dissemination of study results will be accomplished through publication in a peer-reviewed medical journal.

Amendments: Any changes made by the authors from protocol to review will be fully described in the section "Differences between protocol and review"

Contributorship statement: GO and JG conceived the idea for this study. UBN, GO and JG developed the methods and together with AN, AK, LK, WAK, AJ, MJVS, EMT, SM, MC and AA, drafted this protocol.

Competing interests: None of the authors report any competing interests.

Funding: This work was supported by the International Centre for Missing and Exploited Children (no specific grant number).

References

1. National Institute for Health Research. International prospective register of systematic reviews (PROSPERO).Availalbe at https://www.crd.york.ac.uk/prospero/; accessed on 1/31/22.
2. World Health Organization. Responding to children and adolescents who have been sexually abused: WHO clinical guidelines. 2017;Available at: https://apps.who.int/iris/bitstream/handle/10665/259270/9789241550147-eng.pdf;jsessionid=37ADD6616474C995A0D79A2EE83BC1DD?sequence=1; accessed May 12, 2020.
3. World Health Organization. Technical report: W.H.O. guidelines for the health sector response to child maltreatment. 2019;Available at https://www.who.int/violence_injury_prevention/publications/violence/Technical-Report-WHO-Guidelines-for-the-health-sector-response-to-child-maltreatment-2.pdf; accessed on Nov 17, 2020.
4. United Nations, Task Team on the SEA Glossary for the Special Coordinator on Improving the United Nations Response to Sexual Exploitation and Abuse. UN Glossary on sexual exploitation and abuse. 2017;Available at https://hr.un.org/sites/hr.un.org/files/SEA%20Glossary%20%20%5BSecond%20Edition%20-%202017%5D%20-%20English_0.pdf; accessed on Nov 18, 2020.
5. Stoltenborgh M, van Lizendoom MH, Euser EM, Bakermans-Kranenburg MJ. A global perspective on child sexual abuse: Meta-analysis of prevalence around the world. *Child Maltreatment.* 2011;16:79-101.
6. Hailes HP, Yu R, Danese A, Fazel S. Long-term outcomes of childhood sexual abuse: an umbrella review. *The lancet Psychiatry.* 2019;6(10):830-839.
7. Klettke B, Hallford DJ, Clancy E, Mellor DJ, Toumbourou JW. Sexting and Psychological Distress: The Role of Unwanted and Coerced Sexts. *Cyberpsychol Behav Soc Netw.* 2019;22(4):237-242.

For peer review only - http://bmjopen.bmj.com/site/about/guidelines.xhtml

8. Alaggia R, Collin-Vézina D, Lateef R. Facilitators and Barriers to Child Sexual Abuse (CSA) Disclosures: A Research Update (2000-2016). *Trauma Violence Abuse.* 2019;20(2):260-283.
9. Substance Abuse and Mental Health Services Administration. SAMHSA's concept of trauma and guidance for a trauma-informed approach. *Substance Abuse and Mental Health Services Administration.* 2014.
10. Naughton A, Nurmatov U, Van Konijnenburg H, et al. Variability in child protection medical evaluations of suspected physical abuse in four European countries: a vignette study. *Child Abuse Review.* 2018;27(404-418.).
11. Vollmer-Sandholm MJ, Myhre AK, Otterman G, Lydersen S, Stray-Pedersen A. Amongst Norwegian paediatricians there is little agreement in management of cases of suspected child maltreatment. *Acta paediatrica.* 2021;110(2865-2872.).
12. Otterman G, Jalsenius M, Maguire S, Sarkadi A, Janson S. Paediatric approaches to child maltreatment are subject to wide organisational variations across Europe. *106.* 2017;7.
13. Berthold O, Jud A, Jarczok M, Heimann T, Fegert J, Clemens V. Reporting "reasonable suspicion" of child abuse-a survey among physicians and psychotherapists in Germany. *Klinische Padiatrie.* 2021;233(221-225).
14. Marsac ML, Kassam-Adams N, Hildenbrand AK, et al. Implementing a Trauma-Informed Approach in Pediatric Health Care Networks. *JAMA Pediatr.* 2016;170(1):70-77.
15. Broaddus-Shea ET, Scott K, Reijnders M, Amin A. A review of the literature on good practice considerations for initial health system response to child and adolescent sexual abuse. *Child Abuse Negl.* 2019:104225.
16. Kellogg N, Abuse atCoC, Neglect. The Evaluation of Sexual Abuse in Children. *Pediatrics.* 2005;116(2):506-512.
17. Ministry of Health & Family Welfare, Government of INdia. Guidelines & protocols: Medico-legal care for survivors/victims of sexual violence. 2014;Available at https://main.mohfw.gov.in/sites/default/files/953522324.pdf; accessed on Nov 9, 2020.
18. Royal College of Paediatrics and Child Health. *The physical signs of child sexual abuse: an updated evidence-based review and guidance for best practice, 2nd edition.* Vol Available at https://www.rcpch.ac.uk/shop-publications/physical-signs-child-sexual-abuse-evidence-based-review ; accessed on 1/31/22.2015.
19. Scottish Government, Chief Medical Officer Directorate. Supporting children and young people who may have experienced child sexual abuse: clinical pathway for healthcare professionals. 2020;Available at https://www.gov.scot/publications/clinical-pathway-healthcare-professionals-working-support-children-young-people-experienced-child-sexual-abuse/; accessed on 1/31/22.
20. Kerbacher S, Pfeifer M, Riener-Hofer R, et al. Overview of clinical forensic services in various countries of the European Union. . *Forensic Sciences Research.* 2019;5(74-84).
21. CA19106 Multi-Sectoral Responses to Child Abuse and Neglect in Europe: Incidence and Trends.Available at https://www.cost.eu/actions/CA19106/; accessed on Apr 11, 2022.
22. Page MJ, McKenzie JE, Bossuyt PM, et al. The PRISMA 2020 statement: an updated guideline for reporting systematic reviews. *BMJ.* 2021;372:n71.
23. Brouwers M, Kho ME, Browman GP, et al. AGREE II: Advancing guideline develpment, reporting and evaluation in heatlhcare. *Can Med Assoc J.* 2010;182 doi: 10.1503/cmaj.090449:E839-842.
24. AGREE Collaboration. Development and validation of an international appraisal instrument for assessing the quality of clinical practice guidelines: the AGREE project. *Qual Saf Health Care.* 2003;12(1):18-23.
25. Guyatt GH, Oxman AD, Vist GE, et al. GRADE: an emerging consensus on rating quality of evidence and strength of recommendations. *BMJ.* 2008;336(7650):924-926.

For peer review only

Appendix I: Updated search strategy_CSA_Guidelines_SR_MEDLINE

1. Child abuse.mp. or Child Abuse/
2. Abused child.mp.
3. Child sexual abuse.mp. or Child Abuse, Sexual/
4. Child sexual exploitation.mp.
5. (sexual violence or sexual assault or rape or sex trafficking or sexual coercion).mp.
6. or/1-5
7. Diagnosis/ or Diagnosis, Differential/ or diagnosis.mp.
8. diagnostic workup.mp.
9. investigation.mp.
10. (management or management tool or medical management or assessment).mp.
11. (critical pathway or clinical pathway or care path or critical path or toolkit).mp.
12. or/7-11
13. (teen or teens or teenage*).mp.
14. (adolesc* or preadolesc* or pre-adolesc* or juvenil*).mp.
15. (youth or youths or youngster*).mp.
16. ((young adj (person* or persons or people)) or early adult*).mp.
17. (student or students or schoolchild*).mp.
18. exp infant/
19. exp Child/
20. Young adult/
21. adolescent/
22. (boy* or girl* or child or children or infant or infants or kid or kids).mp.
23. (pediatri* or paediatri*).mp
24. or/13-23
25. Guideline Adherence/ or guidelines.mp. or Guideline/ or Practice Guideline/
26. clinical guideline.mp.
27. recommendation.mp.
28. recommendation*.mp.
29. guideline*.mp.
30. clinical gudeline*.mp.
31. or/25-30
32. 6 and 12 and 24 and 31
33. limit 32 to yr="2012-2022"

For peer review only

BMJ Open

Appraisal of Published Guidelines in European Countries Addressing the Clinical Care of Childhood Sexual Abuse: Protocol for a Systematic Review.

Journal:	*BMJ Open*
Manuscript ID	bmjopen-2022-064008.R1
Article Type:	Protocol
Date Submitted by the Author:	29-Nov-2022

Complete List of Authors:	Otterman, Gabriel; Linköping University, Department of Biomedical and Clinical Sciences<br>Nurmatov, Ulugbek; Division of Population Medicine, School of Medicine, Cardiff University<br>Naughton, Aideen; Cardiff University,<br>Kemp, Alison; Department of Child Health, Division of Population Medicine<br>Korhonen, Laura; Linköping University, Biomedical and Clinical Sciences<br>Karst, Wouter; Nederlands Forensisch Instituut, Forensic Medicine<br>Jud, Andreas; University Hospital Ulm<br>Vollmer Sandholm, Mary Jo; Oslo University Hospital<br>Mora-Theuer, Eva; Medical University of Vienna<br>Moultrie, Sarah; University of California San Francisco<br>Akhlaq, Ather; Institute of Business Management ,<br>Chalumeau, Martin; Necker-Enfants Malades Hospitals, Department of General Pediatrics and Pediatric Infectious Diseases<br>Greenbaum, Jordan; International Centre for Missing & Exploited Children

**Primary Subject Heading**:	Paediatrics
Secondary Subject Heading:	Public health, Emergency medicine, Evidence based practice, Legal and forensic medicine, Mental health
Keywords:	Child protection < PAEDIATRICS, Protocols & guidelines < HEALTH SERVICES ADMINISTRATION & MANAGEMENT, Quality in health care < HEALTH SERVICES ADMINISTRATION & MANAGEMENT, ACCIDENT & EMERGENCY MEDICINE

SCHOLARONE™
Manuscripts

Major Revisions to Manuscript ID bmjopen-2022-064008 CLEAN

Title: Appraisal of Published Guidelines in European Countries Addressing the Clinical Care of Childhood Sexual Abuse: Protocol for a Systematic Review.

Word count: 1,828

Authors:

Otterman GJ[1], Nurmatov UB[2], Naughton A[2], Kemp A[2], Korhonen L[1], Karst WA[3], Jud A[4], Vollmer Sandholm MJ[5], Mora Theuer EA[6], Moultrie S[7], Akhlaq A[8], Chalumeau M[9], Greenbaum J[10] and the COST Action 19106 Research Team.

Affiliations:
[1]Linköping University, Linköping, Sweden
[2]Cardiff University, Wales, United Kingdom
[3]Netherlands Forensic Institute, The Hague, Netherlands
[4]Ulm University Hospital, Ulm, Germany
[5]Oslo University Hospital, Oslo, Norway
[6]Medical University of Vienna, Vienna, Austria
[7]University of California San Francisco, San Francisco, United States of America
[8]Institute of Business Management, Karachi, Pakistan
[9] Necker-Enfants malades Hospital, Assistance Publique – Hôpitaux de Paris, Université Paris Cité, Paris, France
[10]International Centre for Missing and Exploited Children, Alexandria, United States of America

Corresponding author:

Name of the corresponding author:	Gabriel J. Otterman
Affiliation:	Barnafrid National Centre on Violence Against Children<br>Department of Biomedical and Clinical Sciences<br>Linköping University<br>SE-58183 Linköping<br>Sweden
Email:	gabriel.otterman@liu.se

ABSTRACT:

Introduction: Child sexual abuse (CSA) is a global public health problem with potentially severe health and mental health consequences. Healthcare professionals (HCPs) should be familiar with risk factors and potential indicators of CSA, and able to provide appropriate medical management. The World Health Organization (WHO) issued global guidelines for the clinical care of children with CSA, based on rigorous review of the evidence base. The current systematic review identifies existing CSA guidelines issued by government agencies and academic societies in the European Region and assesses their quality and clarity to illuminate strengths and identify opportunities for improvement.

Methods and analysis: This 10-database systematic review will be conducted according to the Centre for Reviews and Dissemination guidelines and will be reported according to The Preferred Reporting Items for Systematic reviews and Meta-Analyses (PRISMA) statement. Guidance for HCPs regarding CSA, written by a national governmental agency or academic society of HCPs within 34 COST Action 19106

For peer review only - http://bmjopen.bmj.com/site/about/guidelines.xhtml

Network Countries (CANC) and published in peer-reviewed or grey literature between January 2012 to November 2022 are eligible for inclusion. Two independent researchers will search the international literature, screen, review and extract data. Included guidelines will be assessed for completeness and clarity, compared to the WHO 2017/2019 guidelines on CSA, and evaluated for consistency between the CANC guidelines. The Appraisal of Guidelines for Research and Evaluation (AGREE II) tool and Grading of Recommendations Assessment, Development, and Evaluation (GRADE) methodology will be used to evaluate CANC guidelines. Descriptive statistics will summarise content similarities and differences between the WHO guidelines and national guidelines; data will be summarised using counts, frequencies, proportions and percent agreement between country-specific guidelines and the WHO 2017 guidelines.

Ethics and dissemination: There are no individuals or protected health information involved and no safety issues identified. Results will be published in a peer-reviewed medical journal.

Registration details: PROSPERO #: CRD42022320747.

Strengths and Limitations:

Strengths:

- This systematic review will be the first in this field of research ever undertaken that evaluate the quality and consistency of CSA clinical guidelines across European countries.
- An international team of subject-matter experts in the field of child maltreatment will conduct this study with experts in systematic review methodology using a robust and transparent approach.
- For the comparison of CSA Clinical Guidelines in CANC countries, a reference standard – the WHO evidence-based guidelines - will be employed.

Limitations:

- The different methods used in developing the guidelines across a diversity of practice settings may underpin the variability of both scope and quality of guidelines.
- Our search strategy that is limited in scope to a national level may miss guidelines that are used in practice in European countries where healthcare is organised at the state, provincial or municipal level.

INTRODUCTION

Rationale:

Childhood sexual abuse (CSA), as defined by the United Nations and the World Health Organization (WHO), encompasses a spectrum of inappropriate and (potentially) criminal sexual activities involving children and youth under 18 years of age.[1,2] Rates of CSA are generally high across the globe, with prevalence estimates ranging from 3.7% to 40% in European countries.[3] Adverse physical health, mental health and societal consequences of CSA include infection (e.g., HIV, syphilis and other sexually transmitted infections [STIs]); anogenital injury; unwanted pregnancy; substance abuse disorders; post-traumatic stress disorder (PTSD); major depression, often associated with self-harm and suicidal ideation; anxiety disorders and behavioural challenges, including antisocial behavior.[4,5] As a result of these health conditions, many children and youth who experience one or more forms of CSA seek care from health

For peer review only - http://bmjopen.bmj.com/site/about/guidelines.xhtml

professionals. They may present for acute care after a specific event or may seek care for other reasons which may or may not be related to their victimisation. In the latter case, patients may not disclose their status and the healthcare professional (HCP) must be alert to potential risk factors and indicators of CSA.[6] In addition, the clinician must be knowledgeable about the appropriate set of responses in the care and follow-up of CSA and skilled at providing care using a trauma-informed, patient-centred approach.[7] Training of HCPs on CSA is often lacking in Europe.[8-11] Similarly, medical and nursing students are not routinely taught about trauma-informed, patient-centred care, which involves recognising the potential impact of prior traumatising events on a patient, caregiver and others, and responding with empathy and support. With a trauma-informed approach the HCP makes every effort to minimize patient and caregiver distress during the visit, to actively involve these individuals in discussions and decisions, to prioritise their needs and facilitate short and long-term healing. [12]

To assist HCPs in recognising and appropriately responding to CSA, healthcare organisations need specific protocols and guidelines providing detailed information that aims to optimise care and maximise efficiency. Based upon rigorous review of the global scientific literature on childhood sexual violence,[13] the World Health Organization (WHO) published evidence-based, comprehensive clinical guidelines in 2017 and 2019.[2,14] Countries may rely on these documents to guide their HCPs or base their national guidelines on WHO content. National and regional medical academic societies also publish guidelines.[15-19] However, the nature of CSA guidelines varies in content and detail. HCPs in many countries do not have access to guidelines addressing CSA, due to lack of resources at the local, national or geographic regional level and lack of awareness of the WHO guidelines. This may lead to differences across countries and regions in access to evidence-based medical care for vulnerable children and youth.

It is important to identify existing CSA guidelines issued by government agencies and academic societies in the European Region and assess their quality and clarity in order to illuminate strengths and identify opportunities for improvement. This will help drive a targeted approach to enhancing the healthcare response to CSA and facilitate comprehensive protocol development across the European Region. We posit that disparities in healthcare and health-related outcomes for survivors will decrease as quality of care is increased across the European countries.

Objectives:

The aim of this systematic review is to critically appraise existing clinical guidelines addressing the clinical care of CSA published in the COST Action 19106 Network Countries (CANC).[20] The review will address the following questions:

1. In comparison to the World Health Organization's 2017 and 2019 clinical guidelines on the health sector response to child sexual abuse, what are the completeness, clarity and consistency of healthcare guidelines published by countries within the European Region addressing CSA?
2. How do the included CANC countries' guidelines compare with one another in content consistency, and what discrepancies are manifest in the comparison between countries?

METHODS

This systematic review will be conducted according to the Centre for Reviews and Dissemination guidelines and will be reported according to guidance from the Preferred Reporting Items for Systematic reviews and Meta-Analyses (PRISMA) statement.[21]

For peer review only - http://bmjopen.bmj.com/site/about/guidelines.xhtml

Registration: In accordance with PRISMA-P guidelines, our systematic review protocol was registered with the International Prospective Register of Systematic Reviews (PROSPERO)[22] on registration number: (CRD42022320747).

Eligibility criteria: Guidelines will be eligible for inclusion in the systematic review if they meet all the following criteria:

- Include specific guidance for HCPs to recognise and appropriately respond to children (<18 years) who may have experienced CSA
- Are published in peer-reviewed journals or in the grey literature (including government or health professional society websites)
- Represent the product of a national governmental agency or an academic society of health professionals located within the CANC
- Are published between January 2012, through November 2022 (the time period of ten years was determined based on the subject matter experts' familiarity as clinicians with the publication and utilisation of CSA guidelines, and the estimated frequency of updates of guidelines required to maintain their validity in the face of developments in the underlying published evidence [23]. Reports issued prior to this time may be outdated in certain topics such as diagnosis and treatment of infection.)
- No language restrictions

Information sources

Literature search: The systematic review will entail a search of CINAHL, EMBASE, Google Scholar, Guidelines International Network, MEDLINE, PsycINFO, SUMSearch, TRIP, Web of Science, and WHO Global Health Library. References of the guidelines included in the review will be searched for additional guidelines. Search of the grey literature will include the Health Management Information Consortium (HMIC), the National Institute for Health and Care Excellence (NICE, UK), as well as the websites of government health agencies and academic societies within the European Region. In addition, members of the Multi-sectoral Responses to Child Abuse and Neglect in Europe (Euro-CAN; COST Action 19106, a network of experts in child maltreatment and relevant stakeholders representing 34 countries) will be contacted and asked to identify additional relevant guidelines. Paediatricians, forensic physicians, gynaecologists and other physicians in the CANC engaged in the care of vulnerable children will be included in the search using a snowball strategy.

Search strategy: Search terms will be developed under the expert guidance of one of the research team leads (UN) using medical subject headings (MeSH) and text words related to forms of childhood sexual abuse, and healthcare professional guidance. A draft of a MEDLINE search strategy is included in Appendix 1. After finalisation of the MEDLINE search strategy criteria, the latter will be adapted for the remaining electronic databases. The time period and language restrictions for guideline inclusion are described above.

Patient and public involvement statement: Patients and the public were not involved in this systematic review of published national guidelines.

Study period for the systematic review: The study commenced in May 2022 and completion is anticipated in May 2023.

Study records

For peer review only - http://bmjopen.bmj.com/site/about/guidelines.xhtml

Data management: Retrieved studies from all international electronic databases will be imported into EndNote software and de-duplicated, before being screened and identified as eligible guidelines. Completed screening records, and full texts of included studies will be stored as a Master Database in EndNote software for evaluation.

Selection process: Two authors will search databases and screen titles and abstracts independently for potentially eligible studies. Disagreement between researchers will be resolved by consensus or arbitration involving a third author where necessary. Full texts of studies will be retrieved for selected guidelines, and two authors will evaluate whether these meet inclusion/exclusion criteria. Disagreement will be resolved by referral to a third author if necessary. Reasons for the excluded papers will also be provided. The list of final selected studies will be shared with experts (max. 3) for any missing or on-going/unpublished studies.

Data collection process

Two independent reviewers will extract data from included guidelines and enter it into a customised data extraction sheet. The two sheets will be compared for consistency and discrepancies resolved as above. The resultant data will be entered into the master database (Microsoft Excel). Non-English guidelines will be translated using readily available tools such as Google Translate and by consulting individuals who are bilingual, including in that particular language.

Data items: The customised data extraction sheet will include the following information: country, year of dissemination, guideline development process with the report of the group membership involved, search method, and specific content and characteristics of guidelines as they relate to the good practice statements and recommendations from the "Responding to children and adolescents who have been sexually abused: WHO 2017 clinical guidelines"[2] and the sections on identification and prevention of recurrence of CSA included in the "WHO Guidelines for the Health Sector Response to Child Maltreatment: Technical Report, 2019."[14] Examples of content and characteristics to be assessed and extracted include components of trauma-informed care, and of HIV post-exposure prophylaxis.

Outcomes and prioritisation:
Primary outcome(s):
Alignment with WHO guidelines.[2,14] Completeness, clarity and consistency of national guidelines compared to reference standard.
Secondary outcomes:
Consistency between the included clinical guidelines of the CANC.

Risk of bias in individual studies: Eligible guidelines will be critically appraised by two or more independent reviewers working in duplicate. The quality, methodological rigour and transparency of included clinical guidelines will be assessed using Appraisal of Guidelines for Research & Evaluation AGREE II tool.[24,25]

Data synthesis: Descriptive statistics will be employed to characterise content similarities and differences between the reference standard "Responding to children and adolescents who have been sexually abused: WHO 2017 clinical guidelines" and included national guidelines for the diagnosis of child sexual abuse issued in European countries. Key findings from each guideline will be summarised and presented in descriptive summary tables. Data will be reported using counts, frequencies and proportions (e.g., the proportion of guidelines that address a trauma-informed approach). Members of the research team will

For peer review only - http://bmjopen.bmj.com/site/about/guidelines.xhtml

extract key quality indicators from the WHO 2017 guideline. Each country-specific guideline will be examined for the presence of the indicators. The percent agreement on the rating of country-specific guidelines will be calculated and presented. We will summarise the findings by providing a systematic narrative synthesis with the information presented in text and tables.

Meta-biases: No assessment for meta-bias is planned for this protocol review.

Confidence in cumulative estimate: The Grading of Recommendations Assessment, Development, and Evaluation (GRADE) working group methodology will be used to assess the clarity and strength of underlying evidence inside guideline statements. Additionally, the scope description and grade or rating of the guideline recommendations will be assessed.[26]

Ethics and dissemination: The systematic review does not involve individuals or protected health information and there are no safety issues identified. Dissemination of study results will be accomplished through publication in a peer-reviewed medical journal.

Amendments: Any changes made by the authors from protocol to review will be fully described in the section "Differences between protocol and review".

Contributorship statement: GO and JG conceived the idea for this study. UBN, GO and JG developed the methods and together with AN, AK, LK, WAK, AJ, MJVS, EMT, SM, MC and AA, drafted this protocol.

Competing interests: None of the authors report any competing interests.

Funding: This work was supported by the International Centre for Missing and Exploited Children (no specific grant number).

References

1. United Nations, Task Team on the SEA Glossary for the Special Coordinator on Improving the United Nations Response to Sexual Exploitation and Abuse. UN Glossary on sexual exploitation and abuse. 2017;Available at https://hr.un.org/sites/hr.un.org/files/SEA%20Glossary%20%20%5BSecond%20Edition%20-%202017%5D%20-%20English_0.pdf; accessed on Nov 18, 2020.
2. World Health Organization. Responding to children and adolescents who have been sexually abused: WHO clinical guidelines. 2017;Available at: https://apps.who.int/iris/bitstream/handle/10665/259270/9789241550147-eng.pdf;jsessionid=37ADD6616474C995A0D79A2EE83BC1DD?sequence=1; accessed May 12, 2020.
3. Stoltenborgh M, van Lizendoom MH, Euser EM, Bakermans-Kranenburg MJ. A global perspective on child sexual abuse: Meta-analysis of prevalence around the world. *Child Maltreatment.* 2011;16:79-101.
4. Hailes HP, Yu R, Danese A, Fazel S. Long-term outcomes of childhood sexual abuse: an umbrella review. *Lancet Psychiatry.* 2019;6(10):830-839.
5. Klettke B, Hallford DJ, Clancy E, Mellor DJ, Toumbourou JW. Sexting and Psychological Distress: The Role of Unwanted and Coerced Sexts. *Cyberpsychol Behav Soc Netw.* 2019;22(4):237-242.

For peer review only

6. Alaggia R, Collin-Vézina D, Lateef R. Facilitators and Barriers to Child Sexual Abuse (CSA) Disclosures: A Research Update (2000-2016). *Trauma, violence & abuse.* 2019;20(2):260-283.
7. Substance Abuse and Mental Health Services Administration. SAMHSA's concept of trauma and guidance for a trauma-informed approach. *Substance Abuse and Mental Health Services Administration.* 2014.
8. Naughton A, Nurmatov U, Van Konijnenburg H, et al. Variability in child protection medical evaluations of suspected physical abuse in four European countries: a vignette study. *Child Abuse Review.* 2018;27(404-418.).
9. Vollmer-Dandholm MJ, Myhre AK, Otterman G, Lydersen S, Stray-Pedersen A. Amongst Norwegian paediatricians there is little agreement in management of cases of suspected child maltreatment. *Acta paediatrica.* 2021;110(2865-2872.).
10. Otterman G, Jalsenius M, Maguire S, Sarkadi A, Janson S. Paediatric approaches to child maltreatment are subject to wide prganisational variations across Europe. *106.* 2017;7.
11. Berthold O, Jud A, Jarczok M, Heimann T, Fegert J, Clemens V. Reporting "reasonable suspicion" of child abuse-a survey among physicians and psychotherapists in Germany. *Klinische Padiatrie.* 2021;233(221-225).
12. Marsac ML, Kassam-Adams N, Hildenbrand AK, et al. Implementing a Trauma-Informed Approach in Pediatric Health Care Networks. *JAMA Pediatr.* 2016;170(1):70-77.
13. Broaddus-Shea ET, Scott K, Reijnders M, Amin A. A review of the literature on good practice considerations for initial health system response to child and adolescent sexual abuse. *Child abuse & neglect.* 2019:104225.
14. World Health Organization. Technical report: W.H.O. guidelines for the health sector response to child maltreatment. 2019;Available at https://www.who.int/violence_injury_prevention/publications/violence/Technical-Report-WHO-Guidelines-for-the-health-sector-response-to-child-maltreatment-2.pdf; accessed on Nov 17, 2020.
15. Kellogg N, Abuse atCoC, Neglect. The Evaluation of Sexual Abuse in Children. *Pediatrics.* 2005;116(2):506-512.
16. Ministry of Health & Family Welfare, Government of INdia. Guidelines & protocols: Medico-legal care for survivors/victims of sexual violence. 2014;Available at https://main.mohfw.gov.in/sites/default/files/953522324.pdf; accessed on Nov 9, 2020.
17. Royal College of Paediatrics and Child Health. *The physical signs of child sexual abuse: an updated evidence-based review and guidance for best practice, 2nd edition.* Vol Available at https://www.rcpch.ac.uk/shop-publications/physical-signs-child-sexual-abuse-evidence-based-review ; accessed on 1/31/22.2015.
18. Scottish Government, Chief Medical Officer Directorate. Supporting children and young people who may have experienced child sexual abuse: clinical pathway for healthcare professionals. 2020;Available at https://www.gov.scot/publications/clinical-pathway-healthcare-professionals-working-support-children-young-people-experienced-child-sexual-abuse/; accessed on 1/31/22.
19. Kerbacher S, Pfeifer M, Riener-Hofer R, et al. Overview of clinical forensic services in various countries of the European Union. . *Forensic Sciences Research.* 2019;5(74-84).
20. CA19106 Multi-Sectoral Responses to Child Abuse and Neglect in Europe: Incidence and Trends.Available at https://www.cost.eu/actions/CA19106/; accessed on Apr 11, 2022.
21. Page MJ, McKenzie JE, Bossuyt PM, et al. The PRISMA 2020 statement: an updated guideline for reporting systematic reviews. *BMJ.* 2021;372:n71.
22. National Institute for Health Research. International prospective register of systematic reviews (PROSPERO).Availalbe at https://www.crd.york.ac.uk/prospero/; accessed on 1/31/22.

For peer review only - http://bmjopen.bmj.com/site/about/guidelines.xhtml

23. Alderson LJ, Alderson P, Tan TJJoce. Median life span of a cohort of National Institute for Health and Care Excellence clinical guidelines was about 60 months. 2014;67(1):52-55.

24. Brouwers M, Kho ME, Browman GP, et al. AGREE II: Advancing guideline develpment, reporting and evaluation in heatlhcare. *Can Med Assoc J.* 2010;182 doi: 10.1503/cmaj.090449:E839-842.

25. AGREE Collaboration. Development and validation of an international appraisal instrument for assessing the quality of clinical practice guidelines: the AGREE project. *Qual Saf Health Care.* 2003;12(1):18-23.

26. Guyatt GH, Oxman AD, Vist GE, et al. GRADE: an emerging consensus on rating quality of evidence and strength of recommendations. *BMJ.* 2008;336(7650):924-926.

For peer review only

Appendix to revised Manuscript ID bmjopen-2022-064008.R1

Updated search strategy_CSA_Guidelines_SR_MEDLINE format_14_04_2022_Final

1. Child abuse.mp. or Child Abuse/
2. Abused child.mp.
3. Child sexual abuse.mp. or Child Abuse, Sexual/
4. Child sexual exploitation.mp.
5. (sexual violence or sexual assault or rape or sex trafficking or sexual coercion).mp.
6. or/1-5
7. Diagnosis/ or Diagnosis, Differential/ or diagnosis.mp.
8. diagnostic workup.mp.
9. investigation.mp.
10. (management or management tool or medical management or assessment).mp.
11. (critical pathway or clinical pathway or care path or critical path or toolkit).mp.
12. or/7-11
13. (teen or teens or teenage*).mp.
14. (adolesc* or preadolesc* or pre-adolesc* or juvenil*).mp.
15. (youth or youths or youngster*).mp.
16. ((young adj (person* or persons or people)) or early adult*).mp.
17. (student or students or schoolchild*).mp.
18. exp infant/
19. exp Child/
20. Young adult/
21. adolescent/
22. (boy* or girl* or child or children or infant or infants or kid or kids).mp.
23. (pediatri* or paediatri*).mp
24. or/13-23
25. Guideline Adherence/ or guidelines.mp. or Guideline/ or Practice Guideline/
26. clinical guideline.mp.
27. recommendation.mp.
28. recommendation*.mp.
29. guideline*.mp.
30. clinical gudeline*.mp.
31. or/25-30
32. 6 and 12 and 24 and 31
33. limit 32 to yr="2012-2022"

For peer review only

CSA_SR_Search strategy for other databases_free keyword format

(child abuse or child sexual abuse or child sexual exploitation or sexual violence or sexual assault or rape or sex trafficking or sexual coercion)

AND (Diagnosis or diagnostic work up or investigation or management or assessment or clinical pathway or care path or critical pathway)

AND (Infant or child or young adult or adolescent or teenager or boy or girl) AND (Clinical guideline or guideline or practice guideline or recommendation)

BMJ Open

Appraisal of Published Guidelines in European Countries Addressing the Clinical Care of Childhood Sexual Abuse: Protocol for a Systematic Review.

Journal:	*BMJ Open*
Manuscript ID	bmjopen-2022-064008.R2
Article Type:	Protocol
Date Submitted by the Author:	04-Mar-2023
Complete List of Authors:	Otterman, Gabriel; Linköping University, Department of Biomedical and Clinical Sciences<br>Nurmatov, Ulugbek; Division of Population Medicine, School of Medicine, Cardiff University<br>Naughton, Aideen; Cardiff University,<br>Kemp, Alison; Department of Child Health, Division of Population Medicine<br>Korhonen, Laura; Linköping University, Biomedical and Clinical Sciences<br>Jud, Andreas; University Hospital Ulm<br>Vollmer Sandholm, Mary Jo; Oslo University Hospital<br>Mora-Theuer, Eva; Medical University of Vienna<br>Moultrie, Sarah; University of California San Francisco<br>Akhlaq, Ather; Institute of Business Management ,<br>Chalumeau, Martin; Necker-Enfants Malades Hospitals, Department of General Pediatrics and Pediatric Infectious Diseases<br>Karst, Wouter; CGD Branbant Zuidoost, Forensic Medicine<br>Greenbaum, Jordan; International Centre for Missing & Exploited Children
**Primary Subject Heading**:	Paediatrics
Secondary Subject Heading:	Public health, Emergency medicine, Evidence based practice, Legal and forensic medicine, Mental health
Keywords:	Child protection < PAEDIATRICS, Protocols & guidelines < HEALTH SERVICES ADMINISTRATION & MANAGEMENT, Quality in health care < HEALTH SERVICES ADMINISTRATION & MANAGEMENT, ACCIDENT & EMERGENCY MEDICINE

SCHOLARONE™
Manuscripts

Major Revisions (R2) to Manuscript ID bmjopen-2022-064008 CLEAN

Title: Appraisal of Published Guidelines in European Countries Addressing the Clinical Care of Childhood Sexual Abuse: Protocol for a Systematic Review.

Word count: 1959

Authors:

Otterman GJ[1], Nurmatov UB[2], Naughton A[2], Kemp A[2], Korhonen L[1], Jud A[3], Vollmer Sandholm MJ[4], Mora Theuer EA[5], Moultrie S[6], Akhlaq A[7], Chalumeau M[8], Karst WA[9], Greenbaum J[10] and the COST Action 19106 Research Team.

Affiliations:
[1]Linköping University, Linköping, Sweden
[2]Cardiff University, Wales, United Kingdom
[3]Ulm University Hospital, Ulm, Germany
[4]Oslo University Hospital, Oslo, Norway
[5]Medical University of Vienna, Vienna, Austria
[6]University of California San Francisco, San Francisco, United States of America
[7]Institute of Business Management, Karachi, Pakistan
[8]Necker-Enfants malades Hospital, Assistance Publique – Hôpitaux de Paris, Université Paris Cité, Paris, France
[9]GGD Brabant Zuidoost, Eindhoven, Netherlands
[10]International Centre for Missing and Exploited Children, Alexandria, United States of America

Corresponding author:

Name of the corresponding author:	Gabriel J. Otterman
Affiliation:	Barnafrid National Centre on Violence Against Children<br>Department of Biomedical and Clinical Sciences<br>Linköping University<br>SE-58183 Linköping<br>Sweden
Email:	gabriel.otterman@liu.se

ABSTRACT:

Introduction: Child sexual abuse (CSA) is a global public health problem with potentially severe health and mental health consequences. Healthcare professionals (HCPs) should be familiar with risk factors and potential indicators of CSA, and able to provide appropriate medical management. The World Health Organization (WHO) issued global guidelines for the clinical care of children with CSA, based on rigorous review of the evidence base. The current systematic review identifies existing CSA guidelines issued by government agencies and academic societies in the European Region and assesses their quality and clarity to illuminate strengths and identify opportunities for improvement.

Methods and analysis: This 10-database systematic review will be conducted according to the Centre for Reviews and Dissemination guidelines and will be reported according to The Preferred Reporting Items for Systematic reviews and Meta-Analyses (PRISMA) statement. Guidance for HCPs regarding CSA, written by a national governmental agency or academic society of HCPs within 34 COST Action 19106

For peer review only - http://bmjopen.bmj.com/site/about/guidelines.xhtml

Network Countries (CANC) and published in peer-reviewed or grey literature between January 2012 to November 2022 are eligible for inclusion. Two independent researchers will search the international literature, screen, review and extract data. Included guidelines will be assessed for completeness and clarity, compared to the WHO 2017/2019 guidelines on CSA, and evaluated for consistency between the CANC guidelines. The Appraisal of Guidelines for Research and Evaluation (AGREE II) tool and Grading of Recommendations Assessment, Development, and Evaluation (GRADE) methodology will be used to evaluate CANC guidelines. Descriptive statistics will summarise content similarities and differences between the WHO guidelines and national guidelines; data will be summarised using counts, frequencies, proportions and percent agreement between country-specific guidelines and the WHO 2017 guidelines.

Ethics and dissemination: There are no individuals or protected health information involved and no safety issues identified. Results will be published in a peer-reviewed medical journal.

Registration details: PROSPERO #: CRD42022320747.

Strengths and Limitations:

Strengths:

- This systematic review will be the first in this field of research ever undertaken that evaluate the quality and consistency of CSA clinical guidelines across European countries.
- An international team of subject-matter experts in the field of child maltreatment will conduct this study with experts in systematic review methodology using a robust and transparent approach.
- For the comparison of CSA Clinical Guidelines in CANC countries, a reference standard – the WHO evidence-based guidelines - will be employed.

Limitations:

- The different methods used in developing the guidelines across a diversity of practice settings may underpin the variability of both scope and quality of guidelines.
- Our search strategy that is limited in scope to a national level may miss guidelines that are used in practice in European countries where healthcare is organised at the state, provincial or municipal level.

INTRODUCTION

Rationale:

Childhood sexual abuse (CSA), as defined by the United Nations and the World Health Organization (WHO), encompasses a spectrum of inappropriate and (potentially) criminal sexual activities involving children and youth under 18 years of age.[1,2] Rates of CSA are generally high across the globe, with prevalence estimates ranging from 3.7% to 40% in European countries.[3] Adverse physical health, mental health and societal consequences of CSA include infection (e.g., HIV, syphilis and other sexually transmitted infections [STIs]); anogenital injury; unwanted pregnancy; substance abuse disorders; post-traumatic stress disorder (PTSD); major depression, often associated with self-harm and suicidal ideation; anxiety disorders and behavioural challenges, including antisocial behavior.[4,5] As a result of these health conditions, many children and youth who experience one or more forms of CSA seek care from health

For peer review only - http://bmjopen.bmj.com/site/about/guidelines.xhtml

professionals. They may present for acute care after a specific event or may seek care for other reasons which may or may not be related to their victimisation. In the latter case, patients may not disclose their status and the healthcare professional (HCP) must be alert to potential indicators and risk factors of CSA such as extra vulnerabilities to victimization and inequitable access to care among children with disabilities.[6,7] In addition, the clinician must be knowledgeable about the appropriate set of responses in the care and follow-up of CSA and skilled at providing care using a trauma-informed, patient-centred approach.[8] Training of HCPs on CSA is often lacking in Europe.[9-12] Similarly, medical and nursing students are not routinely taught about trauma-informed, patient-centred care, which involves recognising the potential impact of prior traumatising events on a patient, caregiver and others, and responding with empathy and support. With a trauma-informed approach the HCP makes every effort to minimize patient and caregiver distress during the visit, to actively involve these individuals in discussions and decisions, to prioritise their needs and facilitate short and long-term healing. [13]

To assist HCPs in recognising and appropriately responding to CSA, healthcare organisations need specific protocols and guidelines providing detailed information that aims to optimise care and maximise efficiency. Based upon rigorous review of the global scientific literature on childhood sexual violence,[14] the World Health Organization (WHO) published evidence-based, comprehensive clinical practice guidelines (CPGs) in 2017 and 2019.[2,15] In addition, within the reference CPGs, good practice statements on clinical management are included as guidance even when there is a lack of evidence to support specific recommendations. Countries may rely on these documents to guide their HCPs or base their national guidelines on WHO content. National and regional medical academic societies also publish guidelines.[16-20] However, the nature of CSA guidelines varies in content and detail. HCPs in many countries do not have access to guidelines addressing CSA, due to lack of resources at the local, national or geographic regional level and lack of awareness of the WHO guidelines. This may lead to differences across countries and regions in access to evidence-based medical care for vulnerable children and youth.

It is important to identify existing CSA guidelines issued by government agencies and academic societies in the European Region and assess their quality and clarity in order to illuminate strengths and identify opportunities for improvement. This will help drive a targeted approach to enhancing the healthcare response to CSA and facilitate comprehensive protocol development across the European Region. We posit that disparities in healthcare and health-related outcomes for survivors will decrease as quality of care is increased across the European countries.

Objectives:

The aim of this systematic review is to critically appraise existing clinical guidelines addressing the clinical care of CSA published in the COST Action 19106 Network Countries (CANC).[21] The review will address the following questions:

1. In comparison to the World Health Organization's 2017 and 2019 clinical guidelines on the health sector response to child sexual abuse, what are the completeness, clarity and consistency of healthcare guidelines published by countries within the European Region addressing CSA?
2. How do the included CANC countries' guidelines compare with one another in content consistency, and what discrepancies are manifest in the comparison between countries?

METHODS

For peer review only - http://bmjopen.bmj.com/site/about/guidelines.xhtml

This systematic review will be conducted according to the Centre for Reviews and Dissemination guidelines and will be reported according to guidance from the Preferred Reporting Items for Systematic reviews and Meta-Analyses (PRISMA) statement.[22]

Registration: In accordance with PRISMA-P guidelines, our systematic review protocol was registered with the International Prospective Register of Systematic Reviews (PROSPERO)[23] on registration number: (CRD42022320747).

Eligibility criteria: Guidelines will be eligible for inclusion in the systematic review if they meet all the following criteria:

- Include specific guidance - that may include good practice statements – for HCPs to recognise and appropriately respond to children (<18 years) who may have experienced CSA
- Are published in peer-reviewed journals or in the grey literature (including government or health professional society websites)
- Represent the product of a national governmental agency or an academic society of health professionals located within the CANC
- Are published between January 2012, through November 2022 (the time period of ten years was determined based on the subject matter experts' familiarity as clinicians with the publication and utilisation of CSA guidelines, and the estimated frequency of updates of guidelines required to maintain their validity in the face of developments in the underlying published evidence. [24]) Reports issued prior to this time may be outdated in certain topics such as diagnosis and treatment of infection. In addition, the beginning of the study period in January 2012 coincides with the introduction of significant legislation in the European Union in December 2011 of Directive 2011/93 - *Combating the sexual abuse and sexual exploitation of children and child pornography,* which specifies responsibilities of member states to ensure support of victims of CSA.[25])

- No language restrictions

Information sources

Literature search: The systematic review will entail a search of CINAHL, EMBASE, Google Scholar, Guidelines International Network, MEDLINE, PsycINFO, SUMSearch, TRIP, Web of Science, and WHO Global Health Library. References of the guidelines included in the review will be searched for additional guidelines. Search of the grey literature will include the Health Management Information Consortium (HMIC), the National Institute for Health and Care Excellence (NICE, UK), as well as the websites of government health agencies and academic societies within the European Region. In addition, members of the Multi-sectoral Responses to Child Abuse and Neglect in Europe (Euro-CAN; COST Action 19106, a network of experts in child maltreatment and relevant stakeholders representing 34 countries) will be contacted and asked to identify additional relevant guidelines. Paediatricians, forensic physicians, gynaecologists and other physicians in the CANC engaged in the care of vulnerable children will be included in the search using a snowball strategy.

Search strategy: Search terms will be developed under the expert guidance of one of the research team leads (UN) using medical subject headings (MeSH) and text words related to forms of childhood sexual abuse, and healthcare professional guidance. A draft of a MEDLINE search strategy is included in Appendix 1. After finalisation of the MEDLINE search strategy criteria, the latter will be adapted for the remaining electronic databases. The time period and language restrictions for guideline inclusion are described above.

For peer review only - http://bmjopen.bmj.com/site/about/guidelines.xhtml

Patient and public involvement statement: Patients and the public were not involved in this systematic review of published national guidelines.

Study period for the systematic review: The study commenced in May 2022 and completion is anticipated in May 2023.

Study records

Data management: Retrieved studies from all international electronic databases will be imported into EndNote software and de-duplicated, before being screened and identified as eligible guidelines. Completed screening records, and full texts of included studies will be stored as a Master Database in EndNote software for evaluation.

Selection process: Two authors will search databases and screen titles and abstracts independently for potentially eligible studies. Disagreement between researchers will be resolved by consensus or arbitration involving a third author where necessary. Full texts of studies will be retrieved for selected guidelines, and two authors will evaluate whether these meet inclusion/exclusion criteria. Disagreement will be resolved by referral to a third author if necessary. Reasons for the excluded papers will also be provided. The list of final selected studies will be shared with experts (max. 3) for any missing or on-going/unpublished studies.

Data collection process

Two independent reviewers will extract data from included guidelines and enter it into a customised data extraction sheet. The two sheets will be compared for consistency and discrepancies resolved as above. The resultant data will be entered into the master database (Microsoft Excel). Non-English guidelines will be translated using readily available tools such as Google Translate and by consulting individuals who are bilingual, including in that particular language.

Data items: The customised data extraction sheet will include the following information: country, year of dissemination, guideline development process with the report of the group membership involved, search method, and specific content and characteristics of guidelines as they relate to the good practice statements and recommendations from the "Responding to children and adolescents who have been sexually abused: WHO 2017 clinical guidelines"[2] and the sections on identification of CSA included in the "WHO Guidelines for the Health Sector Response to Child Maltreatment: Technical Report, 2019."[15] Examples of content and characteristics to be assessed and extracted include components of trauma-informed care, and of HIV post-exposure prophylaxis.

Outcomes and prioritisation:
Primary outcome(s):
Alignment with WHO guidelines.[2,15] Completeness, clarity and consistency of national guidelines compared to reference standard.
Secondary outcomes:
Consistency between the included clinical guidelines of the CANC.

Risk of bias in individual studies: Eligible guidelines will be critically appraised by two or more independent reviewers working in duplicate. The quality, methodological rigour and transparency of included clinical guidelines will be assessed using Appraisal of Guidelines for Research & Evaluation AGREE II tool.[26,27]

For peer review only - http://bmjopen.bmj.com/site/about/guidelines.xhtml

Data synthesis: Descriptive statistics will be employed to characterise content similarities and differences between the reference standard "Responding to children and adolescents who have been sexually abused: WHO 2017 clinical guidelines" and included national guidelines for the diagnosis of child sexual abuse issued in European countries. Key findings from each guideline will be summarised and presented in descriptive summary tables. Data will be reported using counts, frequencies and proportions (e.g., the proportion of guidelines that address a trauma-informed approach). Members of the research team will extract key quality indicators from the WHO 2017 guideline. Each country-specific guideline will be examined for the presence of the indicators. The percent agreement on the rating of country-specific guidelines will be calculated and presented. In a sub-group analysis, we will compare identified guidelines published in the period from 2012 that preceded publication of the reference WHO guideline in 2017 to those published subsequently. We will summarise the findings by providing a systematic narrative synthesis with the information presented in text and tables.

Meta-biases: No assessment for meta-bias is planned for this protocol review.

Confidence in cumulative estimate: The Grading of Recommendations Assessment, Development, and Evaluation (GRADE) working group methodology will be used to assess the clarity and strength of underlying evidence inside guideline statements. Additionally, the scope description and grade or rating of the guideline recommendations will be assessed.[28]

Ethics and dissemination: The systematic review does not involve individuals or protected health information and there are no safety issues identified. Dissemination of study results will be accomplished through publication in a peer-reviewed medical journal.

Amendments: Any changes made by the authors from protocol to review will be fully described in the section "Differences between protocol and review".

Contributorship statement: GO and JG conceived the idea for this study. UBN, GO and JG developed the methods and together with AN, AK, LK, WAK, AJ, MJVS, EMT, SM, MC and AA, drafted this protocol.

Competing interests: None of the authors report any competing interests.

Funding: This work was supported by the International Centre for Missing and Exploited Children (no specific grant number).

References

1. United Nations, Task Team on the SEA Glossary for the Special Coordinator on Improving the United Nations Response to Sexual Exploitation and Abuse. UN Glossary on sexual exploitation and abuse. 2017;Available at https://hr.un.org/sites/hr.un.org/files/SEA%20Glossary%20%20%5BSecond%20Edition%20-%202017%5D%20-%20English_0.pdf; accessed on Nov 18, 2020.
2. World Health Organization. Responding to children and adolescents who have been sexually abused: WHO clinical guidelines. 2017;Available at: https://apps.who.int/iris/bitstream/handle/10665/259270/9789241550147-

For peer review only - http://bmjopen.bmj.com/site/about/guidelines.xhtml

eng.pdf;jsessionid=37ADD6616474C995A0D79A2EE83BC1DD?sequence=1; accessed May 12, 2020.

3. Stoltenborgh M, van Lizendoom MH, Euser EM, Bakermans-Kranenburg MJ. A global perspective on child sexual abuse: Meta-analysis of prevalence around the world. *Child Maltreatment.* 2011;16:79-101.
4. Hailes HP, Yu R, Danese A, Fazel S. Long-term outcomes of childhood sexual abuse: an umbrella review. *Lancet Psychiatry.* Oct 2019;6(10):830-839.
5. Klettke B, Hallford DJ, Clancy E, Mellor DJ, Toumbourou JW. Sexting and Psychological Distress: The Role of Unwanted and Coerced Sexts. *Cyberpsychol Behav Soc Netw.* Apr 2019;22(4):237-242.
6. Alaggia R, Collin-Vézina D, Lateef R. Facilitators and Barriers to Child Sexual Abuse (CSA) Disclosures: A Research Update (2000-2016). *Trauma, violence & abuse.* Apr 2019;20(2):260-283.
7. Aslam A. *The state of the world's children 2013: children with disabilities.* United Nations Children's Fund (UNICEF); 2013.
8. Substance Abuse and Mental Health Services Administration. SAMHSA's concept of trauma and guidance for a trauma-informed approach. *Substance Abuse and Mental Health Services Administration.* 2014.
9. Naughton A, Nurmatov U, Van Konijnenburg H, et al. Variability in child protection medical evaluations of suspected physical abuse in four European countries: a vignette study. *Child Abuse Review.* 2018;27(404-418.).
10. Vollmer-Dandholm MJ, Myhre AK, Otterman G, Lydersen S, Stray-Pedersen A. Amongst Norwegian paediatricians there is little agreement in management of cases of suspected child maltreatment. *Acta paediatrica.* 2021;110(2865-2872.).
11. Otterman G, Jalsenius M, Maguire S, Sarkadi A, Janson S. Paediatric approaches to child maltreatment are subject to wide prganisational variations across Europe. *106.* 2017;7.
12. Berthold O, Jud A, Jarczok M, Heimann T, Fegert J, Clemens V. Reporting "reasonable suspicion" of child abuse-a survey among physicians and psychotherapists in Germany. *Klinische Padiatrie.* 2021;233(221-225).
13. Marsac ML, Kassam-Adams N, Hildenbrand AK, et al. Implementing a Trauma-Informed Approach in Pediatric Health Care Networks. *JAMA Pediatr.* Jan 2016;170(1):70-77.
14. Broaddus-Shea ET, Scott K, Reijnders M, Amin A. A review of the literature on good practice considerations for initial health system response to child and adolescent sexual abuse. *Child abuse & neglect.* Nov 8 2019:104225.
15. World Health Organization. Technical report: W.H.O. guidelines for the health sector response to child maltreatment. 2019;Available at https://www.who.int/violence_injury_prevention/publications/violence/Technical-Report-WHO-Guidelines-for-the-health-sector-response-to-child-maltreatment-2.pdf; accessed on Nov 17, 2020.
16. Kellogg N, Abuse atCoC, Neglect. The Evaluation of Sexual Abuse in Children. *Pediatrics.* 2005;116(2):506-512.
17. Ministry of Health & Family Welfare, Government of INdia. Guidelines & protocols: Medico-legal care for survivors/victims of sexual violence. 2014;Available at https://main.mohfw.gov.in/sites/default/files/953522324.pdf; accessed on Nov 9, 2020.
18. Royal College of Paediatrics and Child Health. *The physical signs of child sexual abuse: an updated evidence-based review and guidance for best practice, 2nd edition.* Vol Available at https://www.rcpch.ac.uk/shop-publications/physical-signs-child-sexual-abuse-evidence-based-review ; accessed on 1/31/22.2015.

For peer review only

For peer review only - http://bmjopen.bmj.com/site/about/guidelines.xhtml

19. Scottish Government, Chief Medical Officer Directorate. Supporting children and young people who may have experienced child sexual abuse: clinical pathway for healthcare professionals. 2020;Available at https://www.gov.scot/publications/clinical-pathway-healthcare-professionals-working-support-children-young-people-experienced-child-sexual-abuse/; accessed on 1/31/22.
20. Kerbacher S, Pfeifer M, Riener-Hofer R, et al. Overview of clinical forensic services in various countries of the European Union. . *Forensic Sciences Research.* 2019;5(74-84).
21. CA19106 Multi-Sectoral Responses to Child Abuse and Neglect in Europe: Incidence and Trends.Available at https://www.cost.eu/actions/CA19106/; accessed on Apr 11, 2022.
22. Page MJ, McKenzie JE, Bossuyt PM, et al. The PRISMA 2020 statement: an updated guideline for reporting systematic reviews. *BMJ.* 2021;372:n71.
23. National Institute for Health Research. International prospective register of systematic reviews (PROSPERO).Availalbe at https://www.crd.york.ac.uk/prospero/; accessed on 1/31/22.
24. Alderson LJ, Alderson P, Tan TJJoce. Median life span of a cohort of National Institute for Health and Care Excellence clinical guidelines was about 60 months. 2014;67(1):52-55.
25. EU. Consolidated text: Directive 2011/93/EU of the European Parliament and of the Council of 13 December 2011 on combating the sexual abuse and sexual exploitation of children and child pornography, and replacing Council Framework Decision 2004/68/JHA. 2011; https://eur-lex.europa.eu/legal-content/EN/TXT/?uri=CELEX%3A02011L0093-20111217. Accessed March, 2023.
26. Brouwers M, Kho ME, Browman GP, et al. AGREE II: Advancing guideline develpment, reporting and evaluation in heatlhcare. *Can Med Assoc J.* 2010;182 doi: 10.1503/cmaj.090449:E839-842.
27. AGREE Collaboration. Development and validation of an international appraisal instrument for assessing the quality of clinical practice guidelines: the AGREE project. *Qual Saf Health Care.* 2003;12(1):18-23.
28. Guyatt GH, Oxman AD, Vist GE, et al. GRADE: an emerging consensus on rating quality of evidence and strength of recommendations. *BMJ.* 2008;336(7650):924-926.

For peer review only

Appendix to revised Manuscript ID bmjopen-2022-064008.R1

Updated search strategy_CSA_Guidelines_SR_MEDLINE format_14_04_2022_Final

1. Child abuse.mp. or Child Abuse/
2. Abused child.mp.
3. Child sexual abuse.mp. or Child Abuse, Sexual/
4. Child sexual exploitation.mp.
5. (sexual violence or sexual assault or rape or sex trafficking or sexual coercion).mp.
6. or/1-5
7. Diagnosis/ or Diagnosis, Differential/ or diagnosis.mp.
8. diagnostic workup.mp.
9. investigation.mp.
10. (management or management tool or medical management or assessment).mp.
11. (critical pathway or clinical pathway or care path or critical path or toolkit).mp.
12. or/7-11
13. (teen or teens or teenage*).mp.
14. (adolesc* or preadolesc* or pre-adolesc* or juvenil*).mp.
15. (youth or youths or youngster*).mp.
16. ((young adj (person* or persons or people)) or early adult*).mp.
17. (student or students or schoolchild*).mp.
18. exp infant/
19. exp Child/
20. Young adult/
21. adolescent/
22. (boy* or girl* or child or children or infant or infants or kid or kids).mp.
23. (pediatri* or paediatri*).mp
24. or/13-23
25. Guideline Adherence/ or guidelines.mp. or Guideline/ or Practice Guideline/
26. clinical guideline.mp.
27. recommendation.mp.
28. recommendation*.mp.
29. guideline*.mp.
30. clinical gudeline*.mp.
31. or/25-30
32. 6 and 12 and 24 and 31
33. limit 32 to yr="2012-2022"

CSA_SR_Search strategy for other databases_free keyword format

(child abuse or child sexual abuse or child sexual exploitation or sexual violence or sexual assault or rape or sex trafficking or sexual coercion)

AND (Diagnosis or diagnostic work up or investigation or management or assessment or clinical pathway or care path or critical pathway)

AND (Infant or child or young adult or adolescent or teenager or boy or girl) AND (Clinical guideline or guideline or practice guideline or recommendation)

For peer review only

BMJ Open

Appraisal of Published Guidelines in European Countries Addressing the Clinical Care of Childhood Sexual Abuse: Protocol for a Systematic Review.

Journal:	*BMJ Open*
Manuscript ID	bmjopen-2022-064008.R3
Article Type:	Protocol
Date Submitted by the Author:	23-Mar-2023
Complete List of Authors:	Otterman, Gabriel; Linköping University, Department of Biomedical and Clinical Sciences<br>Nurmatov, Ulugbek; Division of Population Medicine, School of Medicine, Cardiff University<br>Akhlaq, Ather; Institute of Business Management ,<br>Naughton, Aideen; Cardiff University,<br>Kemp, Alison; Department of Child Health, Division of Population Medicine<br>Korhonen, Laura; Linköping University, Biomedical and Clinical Sciences<br>Jud, Andreas; University Hospital Ulm<br>Vollmer Sandholm, Mary Jo; Oslo University Hospital<br>Mora-Theuer, Eva; Medical University of Vienna<br>Moultrie, Sarah; University of California San Francisco<br>Chalumeau, Martin; Necker-Enfants Malades Hospitals, Department of General Pediatrics and Pediatric Infectious Diseases<br>Karst, Wouter; CGD Branbant Zuidoost, Forensic Medicine<br>Greenbaum, Jordan; International Centre for Missing & Exploited Children
**Primary Subject Heading**:	Paediatrics
Secondary Subject Heading:	Public health, Emergency medicine, Evidence based practice, Legal and forensic medicine, Mental health
Keywords:	Child protection < PAEDIATRICS, Protocols & guidelines < HEALTH SERVICES ADMINISTRATION & MANAGEMENT, Quality in health care < HEALTH SERVICES ADMINISTRATION & MANAGEMENT, ACCIDENT & EMERGENCY MEDICINE

SCHOLARONE™ Manuscripts

For peer review only - http://bmjopen.bmj.com/site/about/guidelines.xhtml

Minor Revisions (R3) to Manuscript ID bmjopen-2022-064008 CLEAN

Title: Appraisal of Published Guidelines in European Countries Addressing the Clinical Care of Childhood Sexual Abuse: Protocol for a Systematic Review.

Word count: 1969

Authors:

Otterman GJ[1], Nurmatov UB[2], Akhlaq A[3], Naughton A[2], Kemp A[2], Korhonen L[1], Jud A[4], Vollmer Sandholm MJ[5], Mora Theuer EA[6], Moultrie S[7], Chalumeau M[8], Karst WA[9], Greenbaum J[10] and the COST Action 19106 Research Team.

Affiliations:
[1]Linköping University, Linköping, Sweden
[2]Cardiff University, Wales, United Kingdom
[3]Institute of Business Management, Karachi, Pakistan
[4]Ulm University Hospital, Ulm, Germany
[5]Oslo University Hospital, Oslo, Norway
[6]Medical University of Vienna, Vienna, Austria
[7]University of California San Francisco, San Francisco, United States of America
[8]Necker-Enfants malades Hospital, Assistance Publique – Hôpitaux de Paris, Université Paris Cité, Paris, France
[9]GGD Brabant Zuidoost, Eindhoven, Netherlands
[10]International Centre for Missing and Exploited Children, Alexandria, United States of America

Corresponding author:

Name of the corresponding author:	Gabriel J. Otterman
Affiliation:	Barnafrid National Centre on Violence Against Children<br>Department of Biomedical and Clinical Sciences<br>Linköping University<br>SE-58183 Linköping<br>Sweden
Email:	gabriel.otterman@liu.se

ABSTRACT:

Introduction: Child sexual abuse (CSA) is a global public health problem with potentially severe health and mental health consequences. Healthcare professionals (HCPs) should be familiar with risk factors and potential indicators of CSA, and able to provide appropriate medical management. The World Health Organization (WHO) issued global guidelines for the clinical care of children with CSA, based on rigorous review of the evidence base. The current systematic review identifies existing CSA guidelines issued by government agencies and academic societies in the European Region and assesses their quality and clarity to illuminate strengths and identify opportunities for improvement.

Methods and analysis: This 10-database systematic review will be conducted according to the Centre for Reviews and Dissemination guidelines and will be reported according to The Preferred Reporting Items for Systematic reviews and Meta-Analyses (PRISMA) statement. Guidance for HCPs regarding CSA, written by a national governmental agency or academic society of HCPs within 34 COST Action 19106

For peer review only

Network Countries (CANC) and published in peer-reviewed or grey literature between January 2012 to November 2022 are eligible for inclusion. Two independent researchers will search the international literature, screen, review and extract data. Included guidelines will be assessed for completeness and clarity, compared to the WHO 2017/2019 guidelines on CSA, and evaluated for consistency between the CANC guidelines. The Appraisal of Guidelines for Research and Evaluation (AGREE II) tool and Grading of Recommendations Assessment, Development, and Evaluation (GRADE) methodology will be used to evaluate CANC guidelines. Descriptive statistics will summarise content similarities and differences between the WHO guidelines and national guidelines; data will be summarised using counts, frequencies, proportions and percent agreement between country-specific guidelines and the WHO 2017 guidelines.

Ethics and dissemination: There are no individuals or protected health information involved and no safety issues identified. Results will be published in a peer-reviewed medical journal.

Registration details: PROSPERO #: CRD42022320747.

Strengths and Limitations:

Strengths:

- This systematic review will be the first in this field of research ever undertaken that evaluate the quality and consistency of CSA clinical guidelines across European countries.
- An international team of subject-matter experts in the field of child maltreatment will conduct this study with experts in systematic review methodology using a robust and transparent approach.
- For the comparison of CSA Clinical Guidelines in CANC countries, a reference standard – the WHO evidence-based guidelines - will be employed.

Limitations:

- The different methods used in developing the guidelines across a diversity of practice settings may underpin the variability of both scope and quality of guidelines.
- Our search strategy that is limited in scope to a national level may miss guidelines that are used in practice in European countries where healthcare is organised at the state, provincial or municipal level.

INTRODUCTION

Rationale:

Childhood sexual abuse (CSA), as defined by the United Nations and the World Health Organization (WHO), encompasses a spectrum of inappropriate and (potentially) criminal sexual activities involving children and youth under 18 years of age.[1,2] Rates of CSA are generally high across the globe, with prevalence estimates ranging from 3.7% to 40% in European countries.[3] Adverse physical health, mental health and societal consequences of CSA include infection (e.g., HIV, syphilis and other sexually transmitted infections [STIs]); anogenital injury; unwanted pregnancy; substance abuse disorders; post-traumatic stress disorder (PTSD); major depression, often associated with self-harm and suicidal ideation; anxiety disorders and behavioural challenges, including antisocial behavior.[4,5] As a result of these health conditions, many children and youth who experience one or more forms of CSA seek care from health

For peer review only - http://bmjopen.bmj.com/site/about/guidelines.xhtml

professionals. They may present for acute care after a specific event or may seek care for other reasons which may or may not be related to their victimisation. In the latter case, patients may not disclose their status and the healthcare professional (HCP) must be alert to potential indicators and risk factors of CSA such as extra vulnerabilities to victimization and inequitable access to care among children with disabilities.[6,7] In addition, the clinician must be knowledgeable about the appropriate set of responses in the care and follow-up of CSA and skilled at providing care using a trauma-informed, patient-centred approach.[8] Training of HCPs on CSA is often lacking in Europe.[9-12] Similarly, medical and nursing students are not routinely taught about trauma-informed, patient-centred care, which involves recognising the potential impact of prior traumatising events on a patient, caregiver and others, and responding with empathy and support. With a trauma-informed approach the HCP makes every effort to minimize patient and caregiver distress during the visit, to actively involve these individuals in discussions and decisions, to prioritise their needs and facilitate short and long-term healing. [13]

To assist HCPs in recognising and appropriately responding to CSA, healthcare organisations need specific protocols and guidelines providing detailed information that aims to optimise care and maximise efficiency. Based upon rigorous review of the global scientific literature on childhood sexual violence,[14] the World Health Organization (WHO) published evidence-based, comprehensive clinical practice guidelines (CPGs) in 2017 and 2019.[2,15] In addition, within the reference CPGs, good practice statements on clinical management are included as guidance even when there is a lack of evidence to support specific recommendations. Countries may rely on these documents to guide their HCPs or base their national guidelines on WHO content. National and regional medical academic societies also publish guidelines.[16-20] However, the nature of CSA guidelines varies in content and detail. HCPs in many countries do not have access to guidelines addressing CSA, due to lack of resources at the local, national or geographic regional level and lack of awareness of the WHO guidelines. This may lead to differences across countries and regions in access to evidence-based medical care for vulnerable children and youth.

It is important to identify existing CSA guidelines issued by government agencies and academic societies in the European Region and assess their quality and clarity in order to illuminate strengths and identify opportunities for improvement. This will help drive a targeted approach to enhancing the healthcare recognition of and response to CSA and facilitate comprehensive protocol development across the European Region. We posit that disparities in healthcare and health-related outcomes for survivors will decrease as quality of care is increased across the European countries.

Objectives:

The aim of this systematic review is to critically appraise existing clinical guidelines addressing the clinical care of CSA published in the COST Action 19106 Network Countries (CANC).[21] The review will address the following questions:

1. In comparison to the World Health Organization's 2017 and 2019 clinical guidelines on the health sector recognition of and response to child sexual abuse, what are the completeness, clarity and consistency of healthcare guidelines published by countries within the European Region addressing CSA?
2. How do the included CANC countries' guidelines compare with one another in content consistency, and what discrepancies are manifest in the comparison between countries?

METHODS

For peer review only - http://bmjopen.bmj.com/site/about/guidelines.xhtml

Minor Revisions (R3) to Manuscript ID bmjopen-2022-064008 CLEAN

This systematic review will be conducted according to the Centre for Reviews and Dissemination guidelines and will be reported according to guidance from the Preferred Reporting Items for Systematic reviews and Meta-Analyses (PRISMA) statement.[22]

Registration: In accordance with PRISMA-P guidelines, our systematic review protocol was registered with the International Prospective Register of Systematic Reviews (PROSPERO)[23] on registration number: (CRD42022320747).

Eligibility criteria: Guidelines will be eligible for inclusion in the systematic review if they meet all the following criteria:

- Include specific guidance - that may include good practice statements – for HCPs to recognise and appropriately respond to children (<18 years) who may have experienced CSA
- Are published in peer-reviewed journals or in the grey literature (including government or health professional society websites)
- Represent the product of a national governmental agency or an academic society of health professionals located within the CANC
- Are published between January 2012, through November 2022 (the time period of ten years was determined based on the subject matter experts' familiarity as clinicians with the publication and utilisation of CSA guidelines, and the estimated frequency of updates of guidelines required to maintain their validity in the face of developments in the underlying published evidence. [24]) Reports issued prior to this time may be outdated in certain topics such as diagnosis and treatment of infection. In addition, the beginning of the study period in January 2012 coincides with the introduction of significant legislation in the European Union in December 2011 of Directive 2011/93 - *Combating the sexual abuse and sexual exploitation of children and child pornography,* which specifies responsibilities of member states to ensure support of victims of CSA.[25])

- No language restrictions

Information sources

Literature search: The systematic review will entail a search of CINAHL, EMBASE, Google Scholar, Guidelines International Network, MEDLINE, PsycINFO, SUMSearch, TRIP, Web of Science, and WHO Global Health Library. References of the guidelines included in the review will be searched for additional guidelines. Search of the grey literature will include the Health Management Information Consortium (HMIC), the National Institute for Health and Care Excellence (NICE, UK), as well as the websites of government health agencies and academic societies within the European Region. In addition, members of the Multi-sectoral Responses to Child Abuse and Neglect in Europe (Euro-CAN; COST Action 19106, a network of experts in child maltreatment and relevant stakeholders representing 34 countries) will be contacted and asked to identify additional relevant guidelines. Paediatricians, forensic physicians, gynaecologists and other physicians in the CANC engaged in the care of vulnerable children will be included in the search using a snowball strategy.

Search strategy: Search terms will be developed under the expert guidance of one of the research team leads (UN) using medical subject headings (MeSH) and text words related to forms of childhood sexual abuse, and healthcare professional guidance. A draft of a MEDLINE search strategy is included in Appendix 1. After finalisation of the MEDLINE search strategy criteria, the latter will be adapted for the remaining electronic databases. The time period and language restrictions for guideline inclusion are described above.

For peer review only - http://bmjopen.bmj.com/site/about/guidelines.xhtml

Patient and public involvement statement: Patients and the public were not involved in this systematic review of published national guidelines.

Study period for the systematic review: The study commenced in May 2022 and completion is anticipated in May 2023.

Study records

Data management: Retrieved studies from all international electronic databases will be imported into EndNote software and de-duplicated, before being screened and identified as eligible guidelines. Completed screening records, and full texts of included studies will be stored as a Master Database in EndNote software for evaluation.

Selection process: Two authors will search databases and screen titles and abstracts independently for potentially eligible studies. Disagreement between researchers will be resolved by consensus or arbitration involving a third author where necessary. Full texts of studies will be retrieved for selected guidelines, and two authors will evaluate whether these meet inclusion/exclusion criteria. Disagreement will be resolved by referral to a third author if necessary. Reasons for the excluded papers will also be provided. The list of final selected studies will be shared with experts (max. 3) for any missing or on-going/unpublished studies.

Data collection process

Two independent reviewers will extract data from included guidelines and enter it into a customised data extraction sheet. The two sheets will be compared for consistency and discrepancies resolved as above. The resultant data will be entered into the master database (Microsoft Excel). Non-English guidelines will be translated using readily available tools such as Google Translate and by consulting individuals who are bilingual, including in that particular language.

Data items: The customised data extraction sheet will include the following information: country, year of dissemination, guideline development process with the report of the group membership involved, search method, and specific content and characteristics of guidelines as they relate to the good practice statements and recommendations from the “Responding to children and adolescents who have been sexually abused: WHO 2017 clinical guidelines”[2] and the sections on identification of CSA included in the “WHO Guidelines for the Health Sector Response to Child Maltreatment: Technical Report, 2019.”[15] Examples of content and characteristics to be assessed and extracted include components of trauma-informed care, and of HIV post-exposure prophylaxis.

Outcomes and prioritisation:
Primary outcome(s):
Alignment with WHO guidelines.[2,15] Completeness, clarity and consistency of national guidelines compared to reference standard.
Secondary outcomes:
Consistency between the included clinical guidelines of the CANC.

Risk of bias in individual studies: Eligible guidelines will be critically appraised by two or more independent reviewers working in duplicate. The quality, methodological rigour and transparency of included clinical guidelines will be assessed using Appraisal of Guidelines for Research & Evaluation AGREE II tool.[26,27]

For peer review only - http://bmjopen.bmj.com/site/about/guidelines.xhtml

Data synthesis: Descriptive statistics will be employed to characterise content similarities and differences between the reference standard "Responding to children and adolescents who have been sexually abused: WHO 2017 clinical guidelines" and included national guidelines for the recognition, diagnosis of and response to child sexual abuse issued in European countries. Key findings from each guideline will be summarised and presented in descriptive summary tables. Data will be reported using counts, frequencies and proportions (e.g., the proportion of guidelines that address a trauma-informed approach). Members of the research team will extract key quality indicators from the WHO 2017 guideline. Each country-specific guideline will be examined for the presence of the indicators. The percent agreement on the rating of country-specific guidelines will be calculated and presented. In a sub-group analysis, we will compare identified guidelines published in the period from 2012 that preceded publication of the reference WHO guideline in 2017 to those published subsequently. We will summarise the findings by providing a systematic narrative synthesis with the information presented in text and tables.

Meta-biases: No assessment for meta-bias is planned for this protocol review.

Confidence in cumulative estimate: The Grading of Recommendations Assessment, Development, and Evaluation (GRADE) working group methodology will be used to assess the clarity and strength of underlying evidence inside guideline statements. Additionally, the scope description and grade or rating of the guideline recommendations will be assessed.[28]

Ethics and dissemination: The systematic review does not involve individuals or protected health information and there are no safety issues identified. Dissemination of study results will be accomplished through publication in a peer-reviewed medical journal.

Amendments: Any changes made by the authors from protocol to review will be fully described in the section "Differences between protocol and review".

Contributorship statement: GO and JG conceived the idea for this study. UBN, GO and JG developed the methods and together with AN, AK, LK, WAK, AJ, MJVS, EMT, SM, MC and AA, drafted this protocol.

Competing interests: None of the authors report any competing interests.

Funding: This work was supported by the International Centre for Missing and Exploited Children (no specific grant number).

References

1. United Nations, Task Team on the SEA Glossary for the Special Coordinator on Improving the United Nations Response to Sexual Exploitation and Abuse. UN Glossary on sexual exploitation and abuse. 2017;Available at https://hr.un.org/sites/hr.un.org/files/SEA%20Glossary%20%20%5BSecond%20Edition%20-%202017%5D%20-%20English_0.pdf; accessed on Nov 18, 2020.
2. World Health Organization. Responding to children and adolescents who have been sexually abused: WHO clinical guidelines. 2017;Available at: https://apps.who.int/iris/bitstream/handle/10665/259270/9789241550147-

For peer review only - http://bmjopen.bmj.com/site/about/guidelines.xhtml

eng.pdf;jsessionid=37ADD6616474C995A0D79A2EE83BC1DD?sequence=1; accessed May 12, 2020.

3. Stoltenborgh M, van Lizendoom MH, Euser EM, Bakermans-Kranenburg MJ. A global perspective on child sexual abuse: Meta-analysis of prevalence around the world. *Child Maltreatment.* 2011;16:79-101.
4. Hailes HP, Yu R, Danese A, Fazel S. Long-term outcomes of childhood sexual abuse: an umbrella review. *Lancet Psychiatry.* Oct 2019;6(10):830-839.
5. Klettke B, Hallford DJ, Clancy E, Mellor DJ, Toumbourou JW. Sexting and Psychological Distress: The Role of Unwanted and Coerced Sexts. *Cyberpsychol Behav Soc Netw.* Apr 2019;22(4):237-242.
6. Alaggia R, Collin-Vézina D, Lateef R. Facilitators and Barriers to Child Sexual Abuse (CSA) Disclosures: A Research Update (2000-2016). *Trauma, violence & abuse.* Apr 2019;20(2):260-283.
7. Aslam A. *The state of the world's children 2013: children with disabilities.* United Nations Children's Fund (UNICEF); 2013.
8. Substance Abuse and Mental Health Services Administration. SAMHSA's concept of trauma and guidance for a trauma-informed approach. *Substance Abuse and Mental Health Services Administration.* 2014.
9. Naughton A, Nurmatov U, Van Konijnenburg H, et al. Variability in child protection medical evaluations of suspected physical abuse in four European countries: a vignette study. *Child Abuse Review.* 2018;27(404-418.).
10. Vollmer-Dandholm MJ, Myhre AK, Otterman G, Lydersen S, Stray-Pedersen A. Amongst Norwegian paediatricians there is little agreement in management of cases of suspected child maltreatment. *Acta paediatrica.* 2021;110(2865-2872.).
11. Otterman G, Jalsenius M, Maguire S, Sarkadi A, Janson S. Paediatric approaches to child maltreatment are subject to wide prganisational variations across Europe. *106.* 2017;7.
12. Berthold O, Jud A, Jarczok M, Heimann T, Fegert J, Clemens V. Reporting "reasonable suspicion" of child abuse-a survey among physicians and psychotherapists in Germany. *Klinische Padiatrie.* 2021;233(221-225).
13. Marsac ML, Kassam-Adams N, Hildenbrand AK, et al. Implementing a Trauma-Informed Approach in Pediatric Health Care Networks. *JAMA Pediatr.* Jan 2016;170(1):70-77.
14. Broaddus-Shea ET, Scott K, Reijnders M, Amin A. A review of the literature on good practice considerations for initial health system response to child and adolescent sexual abuse. *Child abuse & neglect.* Nov 8 2019:104225.
15. World Health Organization. Technical report: W.H.O. guidelines for the health sector response to child maltreatment. 2019;Available at https://www.who.int/violence_injury_prevention/publications/violence/Technical-Report-WHO-Guidelines-for-the-health-sector-response-to-child-maltreatment-2.pdf; accessed on Nov 17, 2020.
16. Kellogg N, Abuse atCoC, Neglect. The Evaluation of Sexual Abuse in Children. *Pediatrics.* 2005;116(2):506-512.
17. Ministry of Health & Family Welfare, Government of INdia. Guidelines & protocols: Medico-legal care for survivors/victims of sexual violence. 2014;Available at https://main.mohfw.gov.in/sites/default/files/953522324.pdf; accessed on Nov 9, 2020.
18. Royal College of Paediatrics and Child Health. *The physical signs of child sexual abuse: an updated evidence-based review and guidance for best practice, 2nd edition.* Vol Available at https://www.rcpch.ac.uk/shop-publications/physical-signs-child-sexual-abuse-evidence-based-review ; accessed on 1/31/22.2015.

For peer review only - http://bmjopen.bmj.com/site/about/guidelines.xhtml

Minor Revisions (R3) to Manuscript ID bmjopen-2022-064008 CLEAN

19. Scottish Government, Chief Medical Officer Directorate. Supporting children and young people who may have experienced child sexual abuse: clinical pathway for healthcare professionals. 2020;Available at https://www.gov.scot/publications/clinical-pathway-healthcare-professionals-working-support-children-young-people-experienced-child-sexual-abuse/; accessed on 1/31/22.
20. Kerbacher S, Pfeifer M, Riener-Hofer R, et al. Overview of clinical forensic services in various countries of the European Union. . *Forensic Sciences Research.* 2019;5(74-84).
21. CA19106 Multi-Sectoral Responses to Child Abuse and Neglect in Europe: Incidence and Trends.Available at https://www.cost.eu/actions/CA19106/; accessed on Apr 11, 2022.
22. Page MJ, McKenzie JE, Bossuyt PM, et al. The PRISMA 2020 statement: an updated guideline for reporting systematic reviews. *BMJ.* 2021;372:n71.
23. National Institute for Health Research. International prospective register of systematic reviews (PROSPERO).Availalbe at https://www.crd.york.ac.uk/prospero/; accessed on 1/31/22.
24. Alderson LJ, Alderson P, Tan TJJoce. Median life span of a cohort of National Institute for Health and Care Excellence clinical guidelines was about 60 months. 2014;67(1):52-55.
25. EU. Consolidated text: Directive 2011/93/EU of the European Parliament and of the Council of 13 December 2011 on combating the sexual abuse and sexual exploitation of children and child pornography, and replacing Council Framework Decision 2004/68/JHA. 2011; https://eur-lex.europa.eu/legal-content/EN/TXT/?uri=CELEX%3A02011L0093-20111217. Accessed March, 2023.
26. Brouwers M, Kho ME, Browman GP, et al. AGREE II: Advancing guideline develpment, reporting and evaluation in heatlhcare. *Can Med Assoc J.* 2010;182 doi: 10.1503/cmaj.090449:E839-842.
27. AGREE Collaboration. Development and validation of an international appraisal instrument for assessing the quality of clinical practice guidelines: the AGREE project. *Qual Saf Health Care.* 2003;12(1):18-23.
28. Guyatt GH, Oxman AD, Vist GE, et al. GRADE: an emerging consensus on rating quality of evidence and strength of recommendations. *BMJ.* 2008;336(7650):924-926.

For peer review only

Appendix to revised Manuscript ID bmjopen-2022-064008.R1

Updated search strategy_CSA_Guidelines_SR_MEDLINE format_14_04_2022_Final

1. Child abuse.mp. or Child Abuse/
2. Abused child.mp.
3. Child sexual abuse.mp. or Child Abuse, Sexual/
4. Child sexual exploitation.mp.
5. (sexual violence or sexual assault or rape or sex trafficking or sexual coercion).mp.
6. or/1-5
7. Diagnosis/ or Diagnosis, Differential/ or diagnosis.mp.
8. diagnostic workup.mp.
9. investigation.mp.
10. (management or management tool or medical management or assessment).mp.
11. (critical pathway or clinical pathway or care path or critical path or toolkit).mp.
12. or/7-11
13. (teen or teens or teenage*).mp.
14. (adolesc* or preadolesc* or pre-adolesc* or juvenil*).mp.
15. (youth or youths or youngster*).mp.
16. ((young adj (person* or persons or people)) or early adult*).mp.
17. (student or students or schoolchild*).mp.
18. exp infant/
19. exp Child/
20. Young adult/
21. adolescent/
22. (boy* or girl* or child or children or infant or infants or kid or kids).mp.
23. (pediatri* or paediatri*).mp
24. or/13-23
25. Guideline Adherence/ or guidelines.mp. or Guideline/ or Practice Guideline/
26. clinical guideline.mp.
27. recommendation.mp.
28. recommendation*.mp.
29. guideline*.mp.
30. clinical gudeline*.mp.
31. or/25-30
32. 6 and 12 and 24 and 31
33. limit 32 to yr="2012-2022"

CSA_SR_Search strategy for other databases_free keyword format

(child abuse or child sexual abuse or child sexual exploitation or sexual violence or sexual assault or rape or sex trafficking or sexual coercion)

AND (Diagnosis or diagnostic work up or investigation or management or assessment or clinical pathway or care path or critical pathway)

AND (Infant or child or young adult or adolescent or teenager or boy or girl) AND (Clinical guideline or guideline or practice guideline or recommendation)

For peer review only
